# OTUB1 promotes metastasis and serves as a marker of poor prognosis in colorectal cancer

**DOI:** 10.1186/1476-4598-13-258

**Published:** 2014-11-28

**Authors:** Yi Zhou, Jiangxue Wu, Xiang Fu, Wuying Du, Ling Zhou, Xiangqi Meng, Hongyan Yu, Jiaxin Lin, Wen Ye, Jiani Liu, Hui Peng, Ran-yi Liu, Changchuan Pan, Wenlin Huang

**Affiliations:** State Key Laboratory of Oncology in Southern China, Sun Yat-sen University Cancer Center;Collaborative Innovation Center for Cancer Medicine, No. 651 Dongfeng East Road, Guangzhou, 510060 China; Medical Oncology, Sichuan Cancer Hospital and Institute, Second People’s Hospital of Sichuan Province, Chengdu, 614000 PR China; Department of Colorectal and Anal Surgery, the Sixth Affiliated Hospital of Sun Yat-sen University, Guangzhou, PR China; CAS Key Laboratory of Pathogenic Microbiology and Immunology, Institute of Microbiology, Chinese Academy of Sciences, Beijing, PR China; Guangdong Provincial Key Laboratory of Tumor-targeted Drug and Guangzhou Enterprise Key Laboratory of Gene Medicine, Guangzhou Doublle Bioproducts Inc., Guangzhou, Guangdong China

**Keywords:** OTUB1, Colorectal cancer, Metastasis, EMT, Prognostic factor

## Abstract

**Background:**

OTUB1 (OTU deubiquitinase, ubiquitin aldehyde binding 1) is a deubiquitinating enzyme (DUB) that belongs to the OTU (ovarian tumor) superfamily. The aim of this study was to clarify the role of OTUB1 in colorectal cancer (CRC) and to identify the mechanism underlying its function.

**Methods:**

Two hundred and sixty CRC samples were subjected to association analysis of OTUB1 expression and clinicopathological variables using immunohistochemical (IHC) staining. Overexpression of OTUB1 was achieved in SW480 and DLD-1 cells, and downregulation of OTUB1 was employed in SW620 cells. Then, migration and invasion assays were performed, and markers of the epithelial-mesenchymal transition (EMT) were analyzed. In addition, hepatic metastasis models in mice were used to validate the function of OTUB1 in vivo.

**Results:**

OTUB1 was overexpressed in CRC tissues, and the expression level of OTUB1 was associated with metastasis. A high expression level of OTUB1 was also associated with poor survival, and OTUB1 served as an independent prognostic factor in multivariate analysis. OTUB1 also promoted the metastasis of CRC cell lines in vitro and in vivo by regulating EMT.

**Conclusions:**

OTUB1 promotes CRC metastasis by facilitating EMT and acts as a potential distant metastasis marker and prognostic factor in CRC. Targeting OTUB1 may be helpful for the treatment of CRC.

**Electronic supplementary material:**

The online version of this article (doi:10.1186/1476-4598-13-258) contains supplementary material, which is available to authorized users.

## Background

Colorectal cancer (CRC) represents a leading cause of cancer mortality worldwide. In the United States, CRC is the third most common cancer among both men and women and was the third leading cause of cancer death in 2012. The five-year survival rates for colon cancer and rectal cancer are 65% and 68%, respectively [1]. The leading cause of death in patients with CRC is liver metastasis; approximately 14-25% of patients with CRC present with liver metastasis at diagnosis, and more patients will develop metastasis after diagnosis [2–4]. Therefore, exploring the molecular makers of metastasis and elucidating the underlying metastatic mechanisms are very important for early diagnosis, intervention, treatment, and prognostic evaluation of patients with CRC.

Deubiquitinating enzymes (DUBs) comprise a large group of proteases that can cleave monoubiquitin or polyubiquitin from target proteins. Many DUBs, such as USP46, USP22, UCHL1, and USP9X, have been shown to play important roles in the proliferation, metastasis, and drug resistance of CRC [5–8]. OTUB1 (OUT deubiquitinase, ubiquitin aldehyde binding 1) belongs to the ovarian tumor domain protease (OTU) family of DUBs [9]. OTUB1 is a cysteine protease that hydrolyses the isopeptide bond between ubiquitin and the target molecule. By recognizing a Lys48-linked ubiquitin chain and inhibiting ubiquitin transfer by binding to UBC13, UBE2D, and UBE2E family E2 enzymes, OTUB1 specifically cleaves Lys48-linked polyubiquitin chains [10–12]. Furthermore, OTUB1 has been reported to inhibit the Lys63-linked polyubiquitin of DNA double-strand breaks by targeting UBC13 [13–15]. OTUB1 has been implicated in the regulation of physiological and pathological processes. For example, OTUB1 regulates T cell anergy via GRAIL [16], and in TGFβ induction, OTUB1 inhibits the ubiquitylation of phospho-SMAD2/3 [17]. OTUB1 also stabilizes c-IAP1 expression and promotes TWEAK-induced activation of the NF-κB and MAPK signaling pathways [18]. P53 is a gatekeeper of cell growth and division, and the important function of OTUB1 is the direct suppression of MDM2-mediated p53 ubiquitination to stabilize and activate p53 [19]. Deletions and mutations of the p53 gene can be detected in 65% to 85% of colorectal tumors [20, 21]. In this study, we investigated the association between OTUB1 expression and survival in CRC and sought to elucidate the molecular mechanisms governing the role of OTUB1 in promoting CRC metastasis.

## Results

### Association between OTUB1 expression and clinicopathological variables in CRC

**Figure 1:**
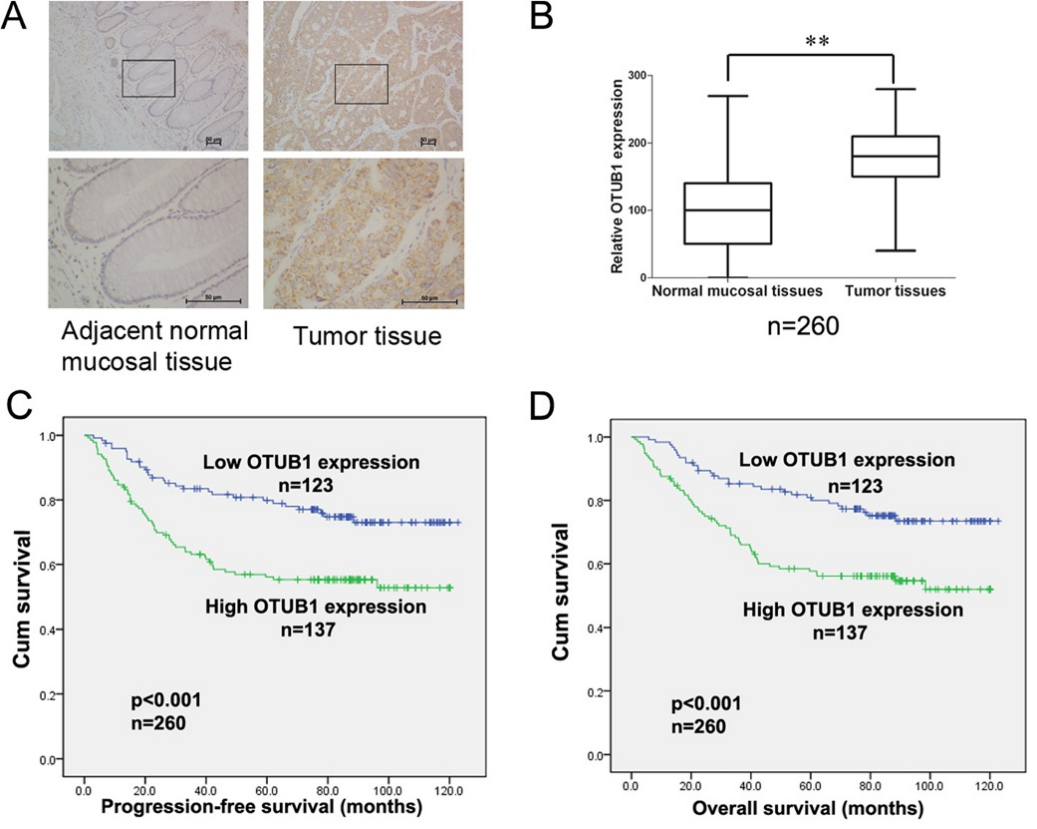
**IHC staining for OTUB1 in 260 CRC tissues. (A)** Representative images (100 and 400 × magnification) of IHC staining for OTUB1 in CRC tissues and paired adjacent normal mucosal tissues. The scale bar represents 50 μm. **(B)** Relative IHC staining for OTUB1 in CRC tissues and adjacent normal mucosal tissues. (n = 260, **P <0.01). **(C)** and **(D)** Kaplan-Meier survival analysis of the correlation between OTUB1 expression and PFS and OS.

To study the potential role of OTUB1 in CRC, we first used IHC staining to analyze the expression of OTUB1 protein in 260 CRC patients who received tumor resection at the Sun Yat-sen University Cancer Center between January 1999 and December 2005. The characteristics of the patients are summarized in Table 1. OTUB1 staining was localized to the cytoplasm. The staining was scored based on the intensity of the staining (4 degrees, Additional file 1: Figure S1) and the proportion of the tumor staining positivity (as described in the Methods). Compared to paired adjacent normal mucosal tissues, the expression of OTUB1 was dramatically higher in tumor tissues (Figure 1A and B, *P* <0.01). High OTUB1 expression was detected in 137 tumor tissues (52.7%), and low OTUB1 expression was observed in 123 tumor samples (47.3%, Table 1).

**Table 1:** **Clinicopathological findings and correlation with OTUB1 expression**

Variables	No. (%)	OTUB1 low	OTUB1 high	P value
**Total cases**	**260**	**123 (47.3)**	**137 (52.7)**	
**Age (years)**				
**<65**	**171 (65.8)**	**79 (30.4)**	**92 (35.4)**	**0.62**
**≥65**	**89 (34.2)**	**44 (16.9 )**	**45 (17.3)**	
**Gender**				
**Male**	**140 (53.8)**	**63 (24.2)**	**77 (29.6)**	**0.421**
**Female**	**120 (46.2)**	**60 (23.1)**	**60 (23.1)**	
**Tumor location**				
**Colon**	**134 (51.5)**	**57 (21.9)**	**77 (29.6)**	**0.112**
**Rectum**	**126 (48.5)**	**66 (25.4)**	**60 (23.1)**	
**Tumor size (cm)**				
**<5**	**117 (45.0)**	**62 (23.8)**	**55 (21.2)**	**0.097**
**≥5**	**143 (55.0)**	**61 (23.5)**	**82 (31.5)**	
**Tumor invasive depth** ^**†**^				
**T1-T2**	**64 (24.6)**	**42 (16.1)**	**22 (8.5)**	0.001*
**T3-T4**	**196 (75.4)**	**81 (31.2)**	**115 (44.2)**	
**Lymph node status**				
**<1**	**140 (53.8)**	**76 (29.2)**	**64 (24.6)**	0.015*
**≥1**	**120 (46.2)**	**47 (18.1)**	**73 (28.1)**	
**Distant metastasis**				
**No metastasis**	**200 (76.9)**	**103 (39.6)**	**97 (37.3)**	0.013*
**metastasis**	**60 (23.1)**	**20 (7.7)**	**40 (15.4)**	
**AJCC/TNM stage**				
**I**	**61 (23.5)**	**40 (15.4)**	**21 (8.1)**	0.003*
**II**	**63 (24.2)**	**30 (11.5)**	**33 (12.7)**	
**III**	**76 (29.2)**	**34 (13.1)**	**42 (16.1)**	
**IV**	**60 (23.1)**	**19 (7.3)**	**41 (15.8)**	
**Chemotherapy**				
**Yes**	**141 (54.2)**	**59 (22.7)**	**82 (31.5)**	**0.055**
**No**	**119 (45.8)**	**64 (24.6)**	**55 (21.2)**	
**Preoperative CEA (ng/mL)** ^**‡**^				
**<=5**	**136 (55.3)**	**71 (28.9)**	**65 (26.4)**	**0.181**
**>5**	**110 (44.7)**	**48 (19.5)**	**62 (25.2)**	

^†^According to the 7th Edition of the AJCC Cancer Staging Manual.

^‡^Analysis for this parameter was available in 246 cases.

*Statistically significant, *P* <0.05.

We next analyzed the relationship between clinicopathological features and the expression level of OTUB1. The results revealed that the expression level of OTUB1 was significantly associated with tumor invasive depth (*P* =0.001), lymph node status (*P* =0.015), distant metastasis (*P* =0.013), and AJCC/TNM stage (*P* =0.003). A high level of OTUB1 expression indicated a greater depth of tumor invasion, the presence of lymph node and distant metastasis. No other significant correlations were observed between the OTUB1 expression level and age, gender, tumor location, tumor size, chemotherapy, or preoperative carcinoembryonic antigen (CEA) expression level (Table 1). Chemotherapy is an important therapy for stage III and IV colorectal cancer patients. We also analyzed the relationship between chemotherapy and the expression level of OTUB1 per stage. As shown in Additional file 2: Table S1, There was no statistical significance.

### OTUB1 overexpression is associated with poor prognosis in CRC

To assess the clinical significance of OTUB1 overexpression in CRC, we analyzed the relationship between the expression level of OTUB1 and patient survival. As shown in Figure 1C and D, OTUB1 expression was negatively associated with PFS (*P* <0.001, HR 2.157, 95% CI, 1.393-3.341) and OS (*P* <0.001, HR 2.187, 95% CI, 1.421-3.387). The five-year rates of PFS (51.1% vs.69.1%) and OS (54.0% vs. 73.1%) were significantly lower in the OTUB1 high-expression group than that in the low-expression group. The subgroup analysis was executed. As shown in Additional file 3: Figure S2, OTUB1 expression was negatively associated with IV stage PFS (*P* <0.016, HR 2.097, 95% CI, 1.138-3.862) and OS (*P* <0.011, HR 2.189, 95% CI, 1.184-4.048). Furthermore, Cox proportional hazards regressions indicated that OTUB1 expression served as an independent prognostic factor for PFS (*P* =0.049, HR 1.61, 95% CI 1.01–2.59) and OS (*P* =0.019, HR 1.77, 95% CI 1.10-2.86; Table 2).

**Table 2:** **Multivariate analysis for PFS and OS**

Variable	PFS		OS	
	HR (95% CI)	p value	HR (95% CI)	p value
**Age (year,<65 vs ≥65)**	**1.29 (0.81-2.07)**	**0. 284**	**1.31 (0.82-2.10)**	**0.256**
**Gender (male vs female)**	**0.77 (0.48-1.22)**	**0.269**	**0.82 (0.50-1.27)**	**0.340**
**Tumor location (colon vs rectum)**	**1.39 (0.88-2.07)**	**0.161**	**1.36 (0.86-2.15)**	**0.184**
**Tumor size (cm, <5 vs ≥5)**	**0.83 (0.48-1.43)**	**0.498**	**0.85 (0.49-1.47)**	**0.553**
**Tumor invasive depth (T1-2 vs T3-4)**	**0.82 (0.33-2.00)**	**0.658**	**0.77 (0.31-1.90)**	**0.567**
**Lymph node status (<1 vs ≥1)**	**2.35 (1.25-4.41)**	0.008*	**2.43 (1.30-4.59)**	0.006*
**Distant metastasis (No vs Yes)**	**7.89 (4.58-13.61)**	<0.001*	**9.41 (5.49-16.16)**	<0.001*
**Preoperative CAE (ng/mL, <5 vs ≥5)**	**1.45 (0.86-2.44)**	**0.160**	**1.54 (0.92-2.56)**	**0.101**
**Chemotherapy (Yes vs no)**	**1.55 (0.94-2.56)**	**0.087**	**1.59 (0.96-2.62)**	**0.069**
**OTUB1 (low vs high)**	**1.61 (1.01-2.59)**	0.049*	**1.77 (1.10-2.86)**	0.019*

PFS progression-free survival, OS overall survival, HR hazard ratio, CI Confidence interval.

*Statistically significant *P* <0.05.

### OTUB1 promotes the migration and invasion of CRC cell lines

Because high OTUB1 expression in primary CRC tissues is associated with lymph node status and distant metastasis, we analyzed whether OTUB1 was highly expressed in lymph node or metastatic tumor tissues. IHC was used to assess the level of OTUB1 expression in 20 grouped samples, including paired adjacent normal mucosal tissues, primary CRC tissues and lymph node or distant metastatic tumor tissues (include 7 liver metastasis, 2 pelvic metastasis and 1ovary metastasis); representative images are shown in Additional file 4: Figure S3A and S3C. OTUB1 expression in lymph node metastatic tumor tissues and primary CRC tissues was higher than that in adjacent normal mucosal tissues (Additional file 4: Figure S3B and Additional file 5: Table S2a). And OTUB1 expression in distant metastatic tumor tissues was dramatically higher than that in adjacent normal mucosal tissues or primary CRC tissues (Additional file 4: Figure S3D and Additional file 5: Table S2b). These results indicated that OTUB1 expression may be associated with CRC metastasis. We therefore analyzed the effect of OTUB1 on the migration and invasion of CRC cells in vitro.

**Figure 2:**
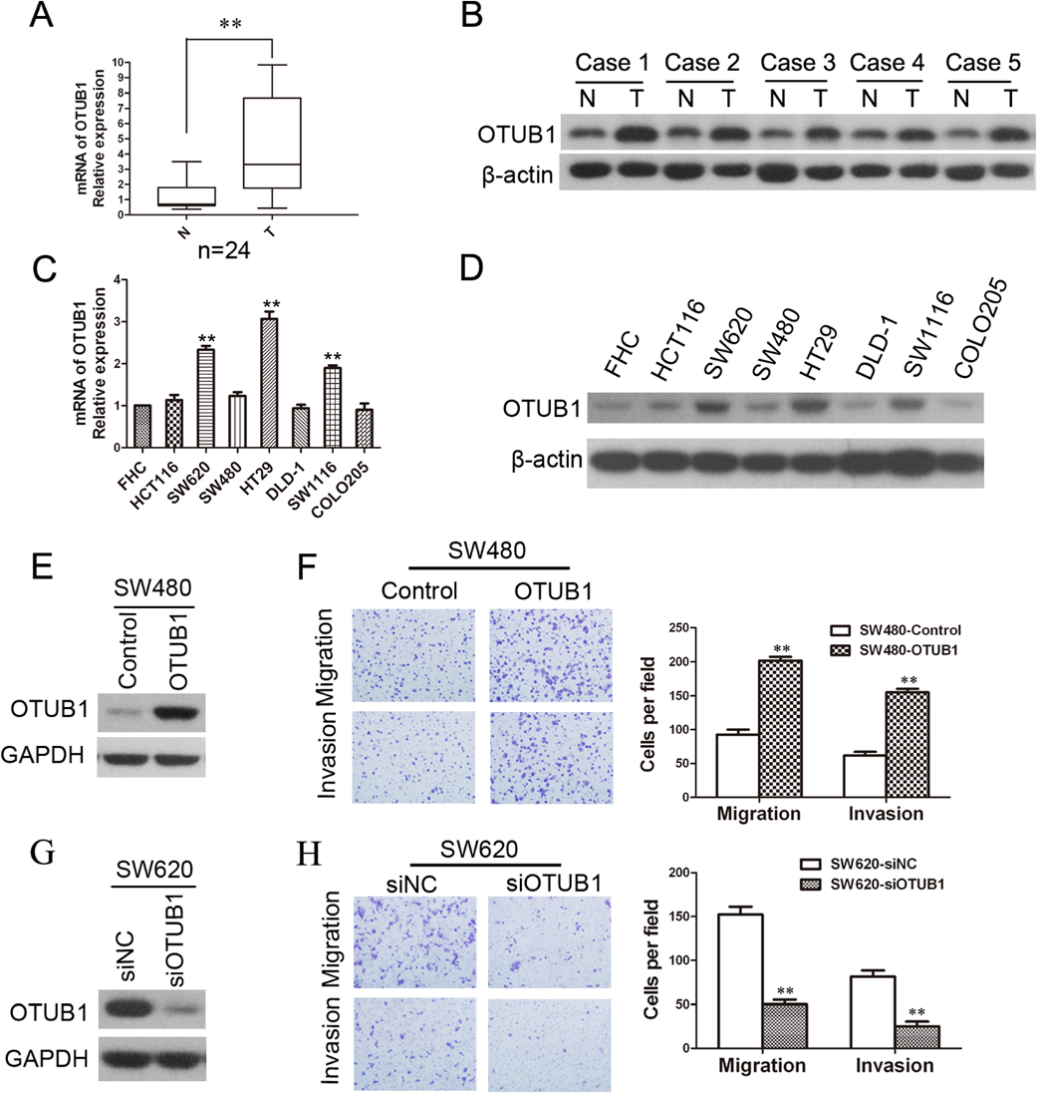
**OTUB1 is expressed in CRC tissues and cell lines and promotes migration and invasion. (A)** The relative expression level of OTUB1 was examined by real-time PCR in 24 CRC tissues (T) and their paired normal mucosal tissues (N). β-actin was used as an endogenous control (**P <0.01). **(B)** OTUB1 expression at the protein level was detected by Western blot analysis in 5 pairs of CRC tissues and paired normal mucosal tissues. β-actin was used as a loading control. The relative mRNA expression level of OTUB1 was examined by real-time PCR **(C)** and the protein level was detected by Western blot analysis **(D)** in 7 CRC cell lines and 1 normal colon epithelial cell line (FHC). β-actin was used as an endogenous control (**P <0.01). **(E)** SW480 cells were transfected with the OTUB1 expression plasmid or empty vector for 48 hours, and the expression of OTUB1 at the protein level was examined by Western blot in the OTUB1 overexpression group (SW480-OTUB1) and the control group (SW480-Control). **(F)** Representative images showing the migration and invasion of SW480-OTUB1 and SW480-Control cells are shown. The number of tumor cells is quantified on the right. All data are expressed as the means of three independent experiments (**P <0.01). **(G)** SW620 cells were transfected with siRNAs against OTUB1 for 48 hours, and the expression level of OTUB1 at the protein level was examined by Western blot. Representative images showing migration and invasion and the quantitative analysis are shown in **(H)**. The data represent the means of three independent experiments, and the error bars represent the SD (**P <0.01).

To investigate the function of OTUB1 in CRC, we examined the expression of OTUB1 in CRC tissues and cell lines. The expression level of mRNA for OTUB1 was increased in 24 CRC tissues compared to their paired normal tissues (Figure 2A, *P* <0.01). The expression of OTUB1 protein was also higher in CRC tissues (Figure 2B). Moreover, the expression of OTUB1 at the mRNA and protein levels was remarkably higher in SW620, HT29 and SW1116 cells compared to FHC cells, and the OTUB1 expression level in HCT116 and SW480 cells was slightly higher than that in FHC cells (Figure 2C and D, *P* <0.01). FHC cell is a normal colon epithelial; HCT116, SW1116 and DLD-1 cells derived from colon primary tumor tissues of patients with colorectal cancer; COLO205 derived from the metastatic site (ascites) of colorectal cancer patient; SW480 derived from a patient with colorectal adenocarcinoma, and SW620 derived from the lymph node of the same patient. So OTUB1 was overexpressed in OTUB1^low^ cell line SW480 and knocked down in OTUB1^high^ cell line SW620 to research the effect of OTUB1 to migration and invasion. After transfecting SW480 cells with OTUB1 expression plasmid or empty vector for 48 hours, we observed that OTUB1 protein was overexpressed in SW480-OTUB1 cells (Figure 2E). Transwell assays showed that the migration and invasion ability of OTUB1-overexpressing cells were enhanced (Figure 2F, *P* <0.01). SiRNAs were also used to knockdown the expression of OTUB1 in SW620 cells, and Western blot analysis showed that OTUB1 was downregulated following siRNA treatment (Figure 2G).The results showed that knockdown of OTUB1 significantly suppressed the migration and invasion of SW620 cells (Figure 2H, *P* <0.01). In additional that we wanted to know whether OTUB1 affect the migration and invasion ability of another cells. Like SW480 and SW620 cells, p53 of DLD-1 cells were mutated, so DLD-1 cells were chose for the model cell. Similar to SW480 cells, after transfecting DLD-1 cells with OTUB1 expression plasmid or empty vector for 48 hours, we observed that OTUB1 protein was overexpressed in DLD-1-OTUB1 cells (Additional file 6: Figure S4A) and the migration and invasion ability was enhanced in DLD-1-OTUB1 cells (Additional file 6: Figure S4B). To investigate whether OTUB1 overexpression or downregulation affected colorectal cells proliferation ability, OTUB1 was transfected in SW480 and DLD1 cells and siRNA of OTUB1 was transfected in SW620 cells, then the cell growth rate was detected by CCK-8. We found OTUB1 did not affect the growth rate of SW480, SW620 and DLD-1 in 4 days (Additional file 7: Figure S5), so the proliferation of cells did not affect the migration and invasion.

### OTUB1 facilitates epithelial-mesenchymal transition (EMT) in CRC cell lines

**Figure 3:**
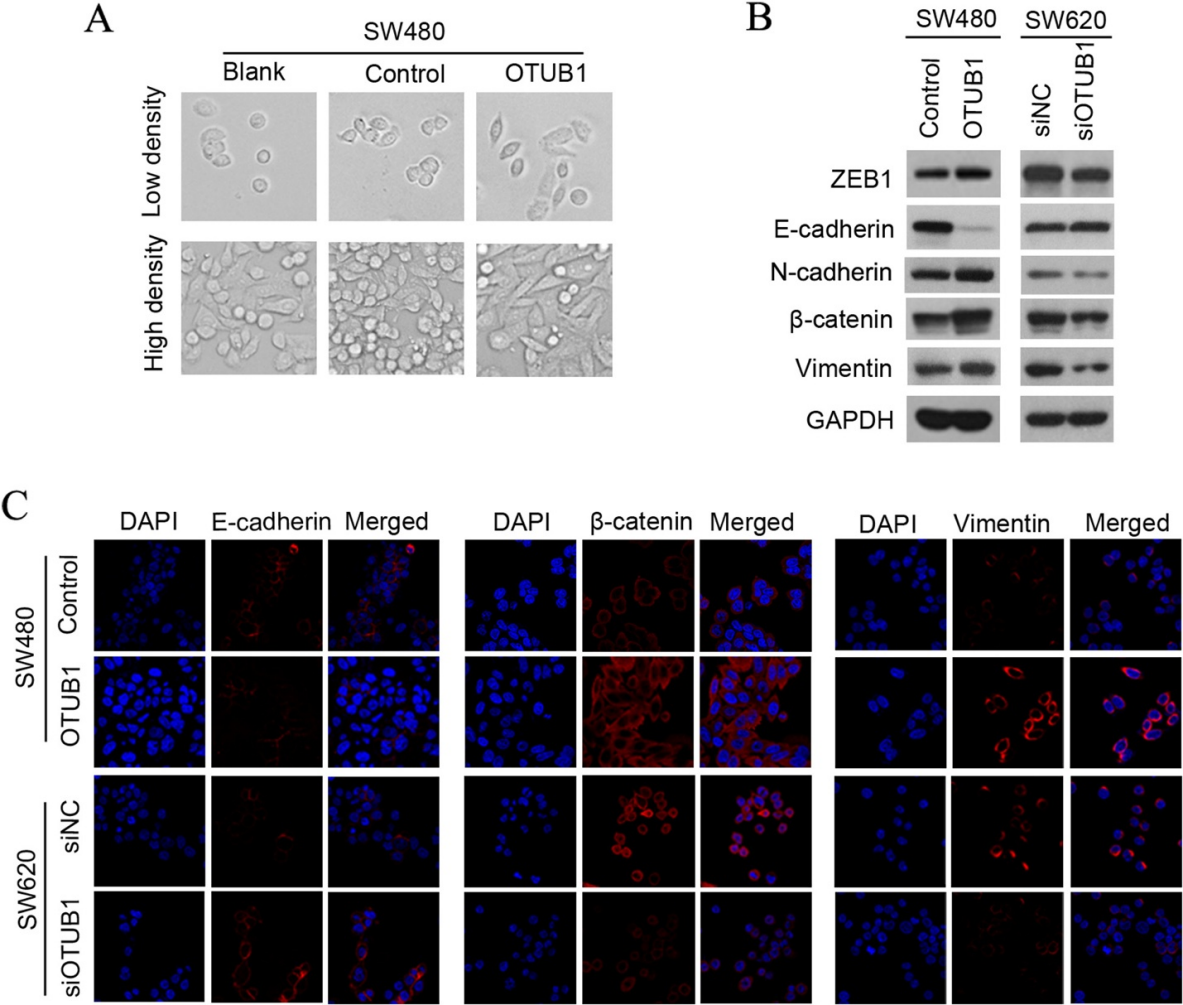
**OTUB1 facilitates EMT in CRC cells. (A)** Morphological change images after overexpressing OTUB1 in SW480. **(B)** After overexpressing OTUB1 in SW480 cells or downregulating OTUB1 expression in SW620 cells, the protein levels of TCF8/ZEB1, E-cadherin, N-cadherin, β-catenin, and vimentin were measured by Western blot. **(C)** Immunofluorescence was used to compare the expression levels and expression pattern of E-cadherin, β-catenin, and vimentin between SW480-OTUB1 and SW480-Control cells or SW620-siOTUB1 and SW620-siNC cells.

Morphological changes were observed in SW480 cells stably expressing OTUB1 (Figure 3A). In particular, the morphology of vector-transfected SW480 cells was similar to that of normal SW480 cells, while OTUB1-overexpressing cells tended to demonstrate an elongated spindle-like shape. EMT is an important factor in cell invasion and morphological changes, so we next evaluated whether EMT markers were altered in our model. The expression of ZEB1, β-catenin, vimentin, E-cadherin, and N-cadherin at the protein level was analyzed by Western blot (Figure 3B). We found that the expression of ZEB1, β-catenin, N-cadherin, and vimentin was increased, whereas E-cadherin expression was decreased, in SW480-OTUB1 cells, and the opposite results were obtained when OTUB1 was knocked down in SW620 cells. Another, the protein expression of vimentin was increased and the protein expression of E-cadherin was decreased in DLD-1-OTUB1 cells (Additional file 8: Figure S6). Furthermore, we performed immunofluorescence analysis to analyze the protein expression of β-catenin, vimentin, and E-cadherin in CRC cell lines (Figure 3C), and these results were consistent with those of the Western blot assays. We also evaluated in the mRNA expression of TCF8/ZEB1, β-catenin, and vimentin in SW480 and SW620 cells, and changes of mRNA expression were consistent with that observed at the protein level (Additional file 9: Figure S7).

β-catenin are important EMT factors that are regulated by GSK3β, which is in turn regulated by the PI3K-AKT signaling pathway. Therefore, we next asked whether the PI3K-AKT-GSK3β signaling pathway was involved in the expression of EMT markers. As shown in Additional file 10: Figure S8A, the overexpression of OTUB1 in SW480 cells decreased PTEN expression and increased p-AKT and p-GSK3β (S9) levels. In contrast, the downregulation of OTUB1 in SW620 cells increased PTEN expression and decreased p-AKT and p-GSK3β (S9) levels. After treatment with LY294002, an inhibitor of the PI3K-AKT signaling pathway, p-AKT and p-GSK3β (S9) levels were decreased in SW480-OTUB1 cells (Additional file 10: Figure S8B), but the expression of E-cadherin, vimentin, and β-catenin was not changed. Therefore, the expression of these EMT markers is not regulated by GSK3β. β-catenin is a important factor in WNT signaling path, so we wanted to know whether OTUB1 affect the activity of WNT signaling path. As shown in Additional file 11: Figure S9, the overexpression of OTUB1 in SW480 and DLD-1 cells or downregulation of OTUB1 in SW620 cells did not changed the protein expression level of TCF1, LEF1 and DVL2. So OTUB1 might be not influence Wnt signaling in our models.

Goncharov reported that the activity of NF-κB and MAKP was regulated by OTUB1 [18], so we evaluated whether activity of NF-κB and MAKP was changed in our models. As shown in Additional file 12: Figure S10, overexpressing OTUB1 in SW480 and DLD-1 cells or downregulating OTUB1 in SW620 cells did not change the expression level of p-JNK, p-p38 (MAPK), p-ERK. So MAPK signal path might not play the major roles in our models. Overexpressing OTUB1 in SW480 and DLD-1 cells did not change the expression level of NF-κB (p65), but it could promote nuclear transference of NF-κB (p65) and activate p-IκBα, and the opposite results were obtained when downregulating OTUB1 in SW620 cells. So the activity of NF-κB was regulated by OTUB1 in our models (Additional file 13: Figure S11).

### Close association between E-cadherin and nuclear β-catenin expression and OTUB1 expression in CRC tissues

**Figure 4:**
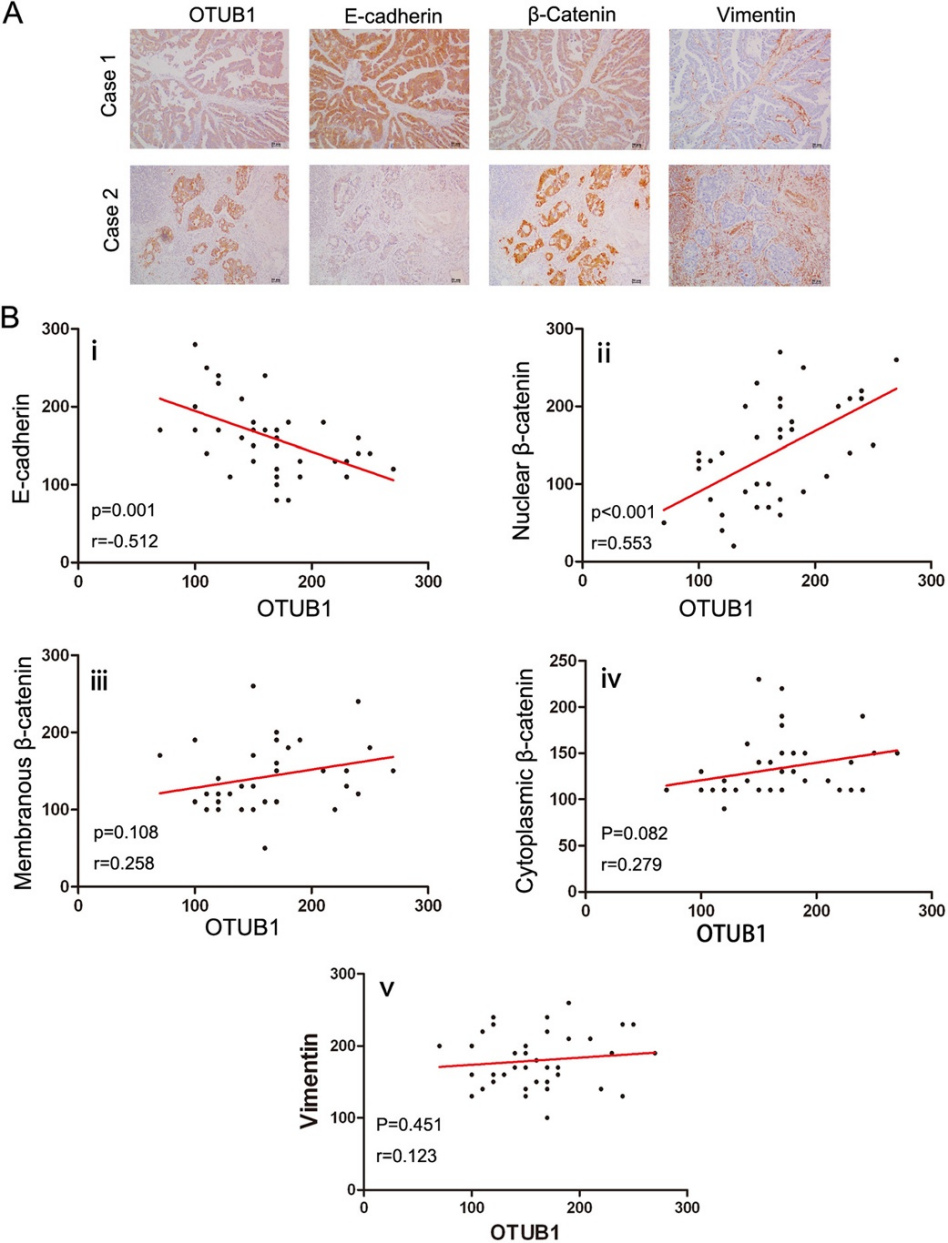
**The correlation between the expression of OTUB1 and E-cadherin, β-catenin, and vimentin in 40 CRC tissues. (A)** Representative IHC staining for OTUB1, E-cadherin, β-catenin, and vimentin is shown in two CRC tissues. The scale bar represents 50 μm. **(B)** A correlation was detected between E-cadherin, β-catenin, vimentin, and OTUB1 expression by Pearson’s test in 40 CRC tissues. The correlations between E-cadherin expression and OTUB1(i), nuclear β-catenin expression and OTUB1(ii), and membranous β-catenin expression and OTUB1(iii) are shown, as well as the correlations between cytoplasmic β-catenin expression and OTUB1(iv) and tumorous stromal vimentin expression and OTUB1(v).

Because OTUB1 was shown to regulate the expression of E-cadherin, β-catenin, and vimentin in CRC cell lines, we sought to determine whether E-cadherin, β-catenin, and vimentin expression was associated with OTUB1 expression in CRC tissues. IHC staining was used to analyze the relationship between the expression of OTUB1 and E-cadherin, β-catenin, and vimentin in 40 CRC tissues; representative images are shown in Figure 4A. E-cadherin expression was negatively correlated with OTUB1 expression (Figure 4B (i), r = -0.512, *P* =0.001). In addition, β-catenin expression was detected in the nucleus, membrane, and cytoplasm of CRC tissues. Nuclear β-catenin expression was positively correlated with OTUB1 expression (Figure 4B (ii), r = 0.553, *P* <0.001). Membranous and cytoplasmic β-catenin expression was not associated with OTUB1 expression (Figure 4B (iii) and (iv)). In CRC tissues, vimentin was expressed in the tumor stroma but was not detected in cancer cells (Figure 4A). The expression of tumorous stromal vimentin was not correlated with OTUB1 expression (Figure 4B (v)).

### Ectopic overexpression of OTUB1 increases the liver metastatic potential of CRC cells in vivo

**Figure 5:**
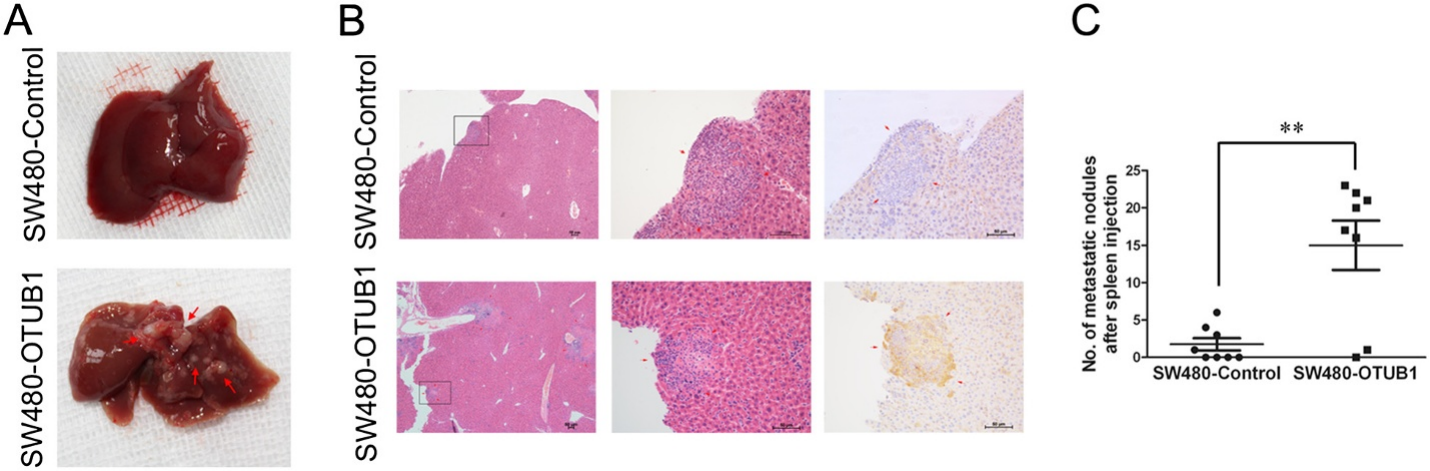
**Overexpression of OTUB1 promotes CRC liver metastasis in vivo.** SW480-OTUB1 or SW480-Control cells were injected into the spleens of nude mice. Ten weeks later, the mice were sacrificed. **(A)** Representative images of general livers after tumor cell injection into the spleen. The metastatic nodules are indicated with red arrows. **(B)** Representative results for HE and IHC staining of metastatic nodules in the livers are shown. The metastatic nodules are indicated with red arrows. The scale bar represents 50 μm. The statistical analyses are shown in **(C)** (n = 8; **P <0.01).

To investigate whether OTUB1 increases the metastatic capacity of CRC cells in vivo, two nude mouse models of liver metastasis were used. SW480-OTUB1 cells, which stably express OTUB1, and SW480-Control cells were injected into the spleens of nude mice. Ten weeks later, the mice were sacrificed. Images showing representative livers with general metastasis were presented in Figure 5A, and images of HE and IHC staining were shown in Figure 5B. In contrast to the SW480-Control group, the expression of OTUB1 was increased and the majority of mice presented liver metastases in the SW480-OTUB1 group, and the number of metastatic nodules in the SW480-OTUB1 group was higher than that observed in the control group (Figure 5C, *P* <0.01).

In another liver metastasis model, SW480-OTUB1 and SW480-Control cells were injected into the tail veins of nude mice. Images showing representative livers with general metastasis and HE and IHC staining are presented in Additional file 14: Figure S12A and S12B. Similar to the spleen injection model, the SW480-OTUB1 group demonstrated more pronounced liver metastasis (Additional file 14: Figure S12C, *P* <0.05).

## Discussion

In this study, we observed that the expression of OTUB1 in CRC tissues was dramatically higher than that in paired normal mucosal tissues (Figure 1A and 1B) and that OTUB1 expression was associated with lymph node status and distant metastasis (Table 1). In particular, high OTUB1 expression in cancer tissues indicated a poorer PFS and OS (Figure 1C and 1D). The subgroup analysis, OTUB1 expression was negatively associated with PFS and OS in stage IV and not associated with stage I, II, III (Additional file 3: Figure S2). There may be a variety of mechanisms involved in OTUB1 promoting the metastasis of colorectal cancer. In our reports, OTUB1 could regulate the expression of EMT markers (Figure 3), PI3K-AKT-GSK3β signaling pathway (Additional file 10: Figure S8B) and nuclear transference of NF-κB (p65) (Additional file 13: Figure S11). In CRC, NF-κB (p65) was significantly higher in primary tumor and liver metastases than normal mucosa. Activation of NF-κB (p65) as measured by nuclear expression is strongly associated with survival and as a prognostic factor in stage IV patients of CRC [22]. So OTUB1 expression may be negatively associated with PFS and OS in stage IV patients in a small specimen. OTUB1 expression was not associated with stage II, III patients in the data, but the number of per tumor stage is small and our results indicated that E-cadherin expression was negatively correlated with OTUB1 expression and nuclear β-catenin expression was positively correlated with OTUB1 expression in colorectal tissues. E-cadherin and nuclear β-catenin are important factors in EMT for CRCs. So we supposed that increasing the number of patients may show the association between OTUB1 expression and stage II and III patients. We will increase the number of patients to research the association between the expression of OTUB1 and per tumor stage in the future. Cox proportional hazards analysis revealed that OTUB1 served as an independent prognostic factor for CRC (Table 2). Chemotherapy is an important treatment for patients with advanced cancer. In our report, chemotherapy was not an independent prognostic factor for CRCs and partial of stage I patients treated with chemotherapy and relatively some stage IV CRC patients did not treat with chemotherapy. The 260 CRC patients in our study were chose between January 1999 and December 2005. And we found that the stage I patients treated with chemotherapy gathered from 1999 to 2003. At that time, the standard treatment of colorectal cancer was not accepted by all doctors in china, so partial of stage I patients were treated with chemotherapy because surgeons worried the recurrent of the patients. And some stage IV CRC patients rejected chemotherapy because they had false beliefs to chemotherapy and did not consider chemotherapy was beneficial for them. But now the standard treatment of colorectal cancer on the basis of NCCN guideline was used in cancer centers in china, such as the Sun Yat-sen University Cancer Center.

More than 25% CRC patients will develop metastasis after diagnosis, and because metastasis is the major cause of death in CRC patients, the identification of prognostic factors for predicting the risk of metastasis is important for improvements in CRC clinical treatment. CEA expression is an established prognostic indicator of postoperative recurrence and metastasis, but this measurement only provides a sensitivity of 34%, a specificity of 84%, and a median lead time of 4.5 months from detection to clinical recurrence [23]. Therefore, other sensitive factors that can predict the metastasis and recurrence of CRC are urgently needed. We found that the expression level of OTUB1 was associated with lymph node status, distant metastasis, and survival in CRC, which suggests that in combination with other markers, OTUB1 may serve as a biomarker of metastasis and a prognostic factor to estimate the prognosis of CRC. However, this study only examined one set of cancer samples from a single clinical center; in the future, we hope to increase the sample size and draw patients from multiple clinical research centers to verify these results.

OTUB1 is an important factor for the stabilization and activation of p53 [19], and the p53 gene is believed to be deleted or mutated in 65% to 85% of CRC patients [20]. In this study, we used SW480 SW620 and DLD-1cells with mutated p53 as models to study the functions of OTUB1 in CRC. We found that overexpressing OTUB1 in SW480, DLD-1 cells or downregulating OTUB1 in SW620 cells did not influence p53 expression (data not shown). So another mechanism might be involved in the metastasis regulation of OTUB1 in CRC. By facilitating the expression of EMT markers (Figure 3 and Figure 4), OTUB1 promoted CRC cell migration and invasion in vitro (Figure 2). We also used an intrasplenic injection mouse model that mimics liver metastasis through the hepatic portal vein and a tail vein injection model that mimics liver metastasis from the systemic circulation. These results revealed that OTUB1 promoted CRC cell liver metastasis in vivo (Figure 5 and Additional file 14: Figure S12). EMT is crucial for the dissemination and invasion of cancers with epithelial origins [24]. The most important event in EMT is the loss of E-cadherin at the cell membrane surface, which is a key hallmark of EMT [25, 26]. The loss of E-cadherin has been detected in various tumors, such as breast carcinoma [27, 28], gastric cancer [29], head and neck cancer [30], lung cancer [31], esophageal cancer [32], and CRC [33, 34]. Previous studies have further shown that the expression of E-cadherin is decreased in colon cancer tissues and in invasive CRC compared to adjacent normal mucosa [35, 36]. The loss of E-cadherin expression results in EMT and cancer metastasis [37], and E-cadherin has been shown to be inhibited by several EMT regulators, including SNAIL, TWIST, ZEB1, and ZEB2. The transcription factor ZEB1, a zinc finger protein encoded by the TCF8 gene, has been shown to act as a transcriptional repressor of E-cadherin [38]. In our reports, E-cadherin expression was regulated by OTUB1 in CRC cell lines, and the level of E-cadherin protein expression was negatively correlated with OTUB1 in CRC tissues (Figure 3 and Figure 4). The expression of ZEB1 was consistent with alterations in OTUB1 expression (i.e., overexpression in SW480 cells or downregulation in SW620 cells), whereas the mRNA and protein expression of E-cadherin was in contrast to that of OTUB1. Together, these results suggest that alterations in E-cadherin expression driven by OTUB1 may be regulated by ZEB1.

Vimentin, a constituent of the intermediate filament family of proteins, is regarded as a canonical marker of EMT. The loss of vimentin has been investigated in many tumor types, including prostate cancer, gastric cancer, lung cancer, malignant melanoma, central nervous system tumors, and CRC [39]. OTUB1 expression in the tumor stroma may also reflect a higher malignant potential of CRC [40, 41]. In present study, vimentin was not expressed in cancer cells but was expressed in tumor stromal cells in CRC tissues, which is in accordance with previous studies. We found that OTUB1 regulated vimentin in CRC cell lines, although vimentin expression in the tumor stromal cells of CRC tissues was not correlated with OTUB1 expression. The reason for this finding may be that the tumor cells of CRC tissues consist of epithelial cells, whereas most of the tumor stromal cells are mesenchymal cells. Vimentin expression is regulated by many factors, such as microRNA-138, which inhibits migration and invasion by directly targeting vimentin in renal cell carcinoma. In addition, the vimentin gene is also highly methylated in CRC tissues [41, 42]. Here, we observed that OTUB1 is a new regulator of vimentin in CRC cell lines.

β-catenin plays a dual role in epithelial cells; this protein is an important component of the adherens junctions linking E-cadherin at the plasma membrane and also acts as the main effector of the canonical WNT signaling cascade in the nucleus [43]. In the nucleus, β-catenin acts as a transcription factor in a complex with the TCF/LEF family of transcription factors [44] and actives downstream genes such as c-myc and cyclin D1. Nuclear β-catenin expression increases from early colorectal adenomas to adenocarcinomas [45], and its high expression has been correlated with lymph node metastasis and poor survival [46]. In our analysis of CRC tissues, the nuclear expression of β-catenin was correlated with OTUB1 expression. This result is consistent with the finding that high OTUB1 expression is associated with poor survival in CRC patients. Moreover, the mRNA and protein expression of β-catenin was found to be regulated by OTUB1, and the changes in β-catenin protein were mostly restricted to the cytoplasm of CRC cell lines (Figure 3C). GSK-3β phosphorylates the N-terminal domain of β-catenin, resulting in the ubiquitylation and degradation of β-catenin [47]. In SW480 cells, GSK3β does not degrade β-catenin due to a lack of APC [48]. At the same time, a high level of N-cadherin expression blocks β-catenin entry into the nucleus [49], so the changes in β-catenin mediated by OTUB1 were observed in the cytoplasm in our model, which might be the reason why TCF1, LEF1 were not activated in our models (Additional file 11: Figure S9). In summary, OTUB1 acts as a multifunctional factor to regulate the expression of E-cadherin, β-catenin, and vimentin during the EMT of CRC cells.

## Conclusions

Together, our findings demonstrate that OTUB1 promotes the migration, invasion, and metastasis of CRC cells in vitro and in vivo and acts as a potential metastasis marker and prognostic factor in CRC. Therefore, it is intriguing to speculate that OTUB1 may be used as a biomarker to predict CRC metastasis and may provide new strategies for treatment.

## Methods

### Immunohistochemical (IHC) staining

CRC tissues were collected at the Sun Yat-sen University Cancer Center. Formalin-fixed, paraffin-embedded cancer tissues were sectioned to a thickness of 4 μm. After routine deparaffinization, rehydration, blocking with hydrogen peroxide, and tissue antigen retrieval with a microwave, the samples were incubated with rabbit anti-OTUB1 polyclonal antibody (ab103995, 1:1,000, Abcam, Cambridge, MA), rabbit anti-β-catenin antibody (#8480, 1:200, Cell Signaling Technology), mouse anti-E-cadherin antibody (#3195, 1:200, Cell Signaling Technology), or rabbit anti-vimentin antibody (#5741, 1:200, Cell Signaling Technology) overnight at 4°C. The slides were stained with secondary antibody and diaminobenzidine tetrahydrochloride (DAKO, Carpinteria, CA) and then counterstained with hematoxylin. The stained slides were evaluated independently by 2 investigators who were unaware of the clinical parameters. The IHC staining was evaluated as described by Abubaker and Cai [50, 51]. The IHC staining intensity was scored from 0-3(I_0_, I_1-3_): negative staining (0), weak staining (1), moderate staining (2), and strong staining (3). The proportion of the tumor staining for that intensity was recorded as 5% increments from a range of 0–100 (P_0_, P_1–3_). The final H score (range 0–300) was calculated for each intensity and proportion of area stained (H score = I_1_XP_1_ + I_2_XP_2_ + I_3_XP_3_). ROC curve analysis was used to determine cutoff value for OTUB1 high expression or OTUB1 low expression. The score that was closest to the point with both maximum sensitivity and specificity was selected as the cutoff value (H score =175).

### Cell lines and culture conditions

The human embryonic kidney cell line HEK-293 T, the human colon cancer cell lines SW480, SW620, HCT116, HT29, DLD-1, SW1116, and COLO205; and the normal colon epithelium cell line FHC were obtained from the American Type Culture Collection. All CRC cell lines and HEK-293 T were cultured in Dulbecco’s modified Eagle’s medium (DMEM) supplemented with 10% fetal bovine serum (FBS). FHC cells were cultured in DMEM:F12 medium (containing 10 ng/ml cholera toxin, 0.005 mg/ml insulin, 0.005 mg/ml transferrin, and 100 ng/ml hydrocortisone) supplemented with 10% FBS. All cells were maintained in a humidified 5% CO_2_ atmosphere at 37°C.

### Real-time PCR analysis ofthe expression of OTUB1

Total RNA was extracted from CRC cell lines and tissues using Trizol (Invitrogen). Two micrograms of total RNA was used to synthesize complimentary DNA with M-MLV reverse transcriptase (Promega, Madison, WI). For quantitative real-time PCR (qPCR), cDNA products were amplified using a SYBR Green PCR Kit (Invitrogen). Quantification was performed using the Stratagene MX300P sequence detection system (Stratagene). OTUB1 expression values were normalized to those of the housekeeping gene β-actin and calculated using the comparative CT method (2^-ΔΔCT^). The primer sequences are provided in Additional file 15: Table S3.

### Cell transfection

To generate the pcDNA3.1-OTUB1 plasmid, full-length human OTUB1 (which was cloned using the primers forward: 5’-ATTGGATCCACCATGGCGGCGGAGGAACCT-3’ and reverse: 5’- ATGCTCGAGCTATTTGTAGAGGATATCGTA-3’) was released by *BamH I* and *Xho I* digestion and inserted into pcDNA3.1. In addition, two siRNAs were designed and synthesized by GenePharma Company to knockdown the expression of OTUB1. The siRNA sequences were as follows: #1, 5’-UUAACTGUCUGGCCUAUGATT-3’ and #2, 5’-CCAUGUGCAAGGAGAGCGATT-3’. A negative siRNA control with the sequence 5’-UUCUCCGAACGUGUCACGUTT-3’ was also used. To perform transient transfections, 4×10^5^ SW480, DLD-1 or SW620 cells were seeded in 6-well plates. Twenty-four hours later, the cells were transfected with 4 μg of plasmid DNA or 100 nM siRNA using Lipofectamine 2000 (Invitrogen, Carlsbad, CA) according to the manufacturer’s protocol. After 48 hours, the cells were collected for qPCR, western blotting, migration, invasion, and immunofluorescence assays**.**

### Western blot analysis

Total cellular proteins were extracted in lysis buffer (1% NP-40, 0.1% sodium dodecyl sulfate, pH 7.3, 50 mM Tris, and 150 mM NaCl) with protease inhibitors (Roche) and phosphatase inhibitors (KeyGEN Biotech) for 30 min on ice and centrifuged at 15,000 rpm at 4°C. Nuclear and Cytoplasmic Protein Extraction kIt (KeyGEN Biotech) was used to separate the nuclear and cytoplasmic protein according to the manufacturer’s protocol. Western blots were carried out as previously described [52]. The primary antibodies used included anti-TCF8/ZEB1, anti-β-catenin, anti-p-GSK3β (S9), anti-GSK3β, anti-AKT, anti-p-AKT, anti-PTEN, anti-E-cadherin, anti-N-cadherin, anti-vimentin, anti-TCF1, anti-LEF1, anti-DVL2, anti-p-JNK, anti-p-ERK, anti-p-p38 (MAPK), anti-NF-κB (p65) , p-IκBα (all 1:1,000; Cell Signaling Technology), anti-OTUB1 (1:1,000; Abcam, Cambridge, MA), anti-GAPDH (1:2,000; Santa Cruz Biotechnology, Santa Cruz, CA), and anti-β-actin (1:2,000; Santa Cruz Biotechnology, Santa Cruz, CA), anti-Histone H3 (1:2,000; Santa Cruz Biotechnology, Santa Cruz, CA).

### Migration and Invasion assays

To study the effect of OTUB1 on the migration and invasion of colorectal cells, 4×10^5^ SW480, DLD-1 or SW620 cells were seeded in 6-well plates. Twenty-four hours later, the cells were transfected with 4 μg of OTUB1 plasmid DNA or 100 nM siRNA using Lipofectamine 2000 according to the manufacturer’s protocol. After transfection with plasmid or siRNA for 24 hours, 1×10^5^ cells in serum-free medium were seeded into the Boyden chamber without Matrigel (8-μm pore; BD Falcon, San Jose, CA) for migration or the chamber with Matrigel (8-μm pore; BD Falcon) for invasion. Then the chambers were put in 24-well plates with medium with 10% FBS. The chambers were incubated for 24 hours at 37°C with 5% CO2. The cells on the underside of filter membrane were fixed in ethanol and stained with crystal violet. The cells were counted under a microscope.

### Measurement of cell proliferation

After transfection with plasmid or siRNA for 6 hours, 3×10^3^ SW480, SW620 and DLD-1 cells were seeded into 96-well culture plates. Cell proliferation was determined using Cell Counting Kit-8 (CCK-8, Dojindo, Kumamoto, Japan) at 0, 24, 48, 72, 96 hours. 10 μL of CCK-8 solution were added to the 90 μL medium and incubated for 2 h in an incubator with 5% CO2 atmosphere at 37°C. The absorbance was measured by a microplate reader at 450 nm.

### Lentivirus packaging and transduction

We used a lentivirus system (System Biosciences, Inc.) to stably express OTUB1 in SW480 cells. OTUB1 cDNA was cloned and inserted into the pCDH- CMV-MCS-EF1 vector. 293 T cells were transfected with pCDH-OTUB1 or pCHD vector and the package plasmids according to the manufacturer’s instructions. Virus particles were harvested 48 hours after transfection. SW480 cells were infected with virus particles and selected by flow cytometry according to green fluorescence. Western blotting was used to detect OTUB1 expression.

### Immunofluorescence

A total of 4×10^5^ cells were seeded into glass-bottom cell culture dishes (NEST Biotechnology Co., LTD). After 24 hours, the cells were fixed in 4% paraformaldehyde, permeabilized using 0.5% Triton X-100, and blocked with 3% bovine serum albumin. The cells were incubated with a primary anti-E-cadherin (1:100, Cell Signaling Technology), anti-β-catenin (1:200, Cell Signaling Technology), or anti-vimentin (1:200, Cell Signaling Technology) antibody overnight at 4°C, followed by incubation with a Alexa fluor-594-conjugated secondary antibody (Invitrogen). The samples were co-stained with 4',6-diamidino-2-phenylindole (DAPI) and examined by confocal microscopy.

### Animal model

Female 4- to 5-week-old BABL/c nude mice were purchased from the Experimental Animal Center of Guangdong Province (Guangzhou, China). All animal studies were conducted in accordance with the current Chinese regulations and standards regarding the use of laboratory animals, and all animal procedures were approved by the Sun Yat-sen University Institutional Animal Care and Use Committee.

The mice were randomly allocated into four groups (n = 8) in two experiments to study liver metastasis. Two groups received spleen injection, and the others received tail vein injection. The experimental procedure has been described previously [53]. For the spleen injection experiment, the mice were anesthetized with isoflurane and laparotomized to pull the spleen out of the abdominal cavity. A total of 1×10^6^ cells (SW480-OTUB1 or SW480-Control) in 20 μl of phosphate-buffered saline (PBS) were slowly injected into the spleen using an insulin syringe. The spleen was then replaced in the abdomen, and the abdominal wall was sutured. Ten weeks later, the mice were euthanized, and the livers were removed for pathological examination. For the tail vein injection experiment, 1.2×10^6^ cells in 100 μl of PBS were injected into the tail vein of mice using an insulin syringe. After ten weeks, the mice were euthanized, and the livers were removed for pathological examination. Formalin-fixed, paraffin-embedded liver tissues were sectioned at a thickness of 5 μm at 10 different planes to cover the entire liver. The sections were stained with hematoxylin and eosin (HE) and IHC, and the metastatic nodules were counted by microscopy [53, 54].

### Statistical analysis

Two-tailed χ2 tests were used to assess the significant associations between OTUB1 expression and clinicopathological parameters. Survival analysis was calculated using the Kaplan-Meier method. The log-rank test was used to compare survival curves. A Cox proportional hazards model was used to calculate the multivariate hazard ratios for clinicopathological parameters and the OTUB1 expression level with respect to overall survival (OS) and progression-free survival (PFS). Pearson’s test was applied for the correlation analysis. The significance of the in vitro and in vivo data was determined using the two-tailed t-test. P-values <0.05 were considered statistically significant. Statistical analyses were performed using the SPSS software package (SPSS Standard version 17.0, SPSS Inc., Chicago, IL).

## Electronic supplementary material

Additional file 1: Figure S1: Representative images (200×magnification) of IHC staining for OTUB1 in CRC tissues. OTUB1 protein expression was scored from 0 to 3. A score of 0 represents negative staining (A), a score of 1 indicates weak positive staining (B), a score of 2 indicates moderate positive staining (C), and a score of 3 represents strong positive staining (D). The scale bar represents 50 μm. (PNG 427 KB)

Additional file 2: Table S1: Chemotherapy of per stage correlation with OTUB1 expression. (DOCX 19 KB)

Additional file 3: Figure S2: Kaplan-Meier survival analysis of the correlation between OTUB1 expression and PFS and OS of per stage. (A) Kaplan-Meier method was used to analyze the correlation between OTUB1 expression and PFS and OS of stage I CRC. (B) Kaplan-Meier method was used to analyze the correlation between OTUB1 expression and PFS and OS of stage II CRC. (C) Kaplan-Meier method was used to analyze the correlation between OTUB1 expression and PFS and OS of stage III CRC. (D) Kaplan-Meier method was used to analyze the correlation between OTUB1 expression and PFS and OS of stage IV CRC. (PNG 2 MB)

Additional file 4: Figure S3: OTUB1 are expressed in 10 paired adjacent normal mucosal tissues, primary tumor tissues and lymph node metastatic tissues or distant metastatic tissues. (A) Representative images of adjacent normal mucosal tissues, primary tumor tissues and lymph node metastatic tumor tissues from one patient sample are shown. (B) Relative IHC staining for OTUB1 in adjacent normal mucosal tissues, primary tumor tissues, and lymph node metastatic tumor tissues is shown (n=10, **P < 0.01). (C) Representative images of adjacent normal mucosal tissues, primary tumor tissues, and distant metastatic tumor tissues from one patient sample are shown. (D) Relative IHC staining for OTUB1 in adjacent normal mucosal tissues, primary tumor tissues, and distant metastatic tissues is shown (n=10, **P < 0.01, * *P* < 0.05). (PNG 3 MB)

Additional file 5: Table S2a: The H scores of OTUB1 expression in normal mucosal tissues, primary tumor tissues and lymph node metastatic tumor tissues in 10 paired tissues. And **Table S2b** The H scores of OTUB1 expression in normal mucosal tissues, primary tumor tissues and distant metastatic tumor tissues in 10 paired tissues. (DOCX 17 KB)

Additional file 6: Figure S4: OTUB1 promotes DLD-1 cells migration and invasion. DLD-1 cells were transfected with the OTUB1 expression plasmid or empty vector for 48 hours, and the expression of OTUB1 at the protein level was examined by Western blot in the OTUB1 overexpression group (DLD-1-OTUB1) and the control group (DLD-1-Control) (A). Representative images showing the migration and invasion of DLD-1-OTUB1 and DLD-1-Control cells are shown (B). The number of tumor cells is quantified on the right. All data are expressed as the means of three independent experiments (**P < 0.01, *P < 0.05). (PNG 1017 KB)

Additional file 7: Figure S5: OTUB1 does not affect the growth of SW480, SW620 and DLD-1. After transfecting SW480, DLD-1 cells with OTUB1 expression plasmid or empty vector or transfecting SW620 cells with siRNA of OTUB1 or NC, the cell growth rate were detected at 0, 24, 48, 72, 96 hours. A-C represented SW480, SW620 and DLD-1 cells respectively. (PNG 949 KB)

Additional file 8: Figure S6: OTUB1 facilitates EMT markers in DLD-1 cells. After overexpressing OTUB1 in DLD-1 cells, the protein levels of E-cadherin, β-catenin, and vimentin were measured by Western blot in the OTUB1 overexpression group (DLD-1-OTUB1) and the control group (DLD-1-Control). (PNG 3 MB)

Additional file 9: Figure S7: The mRNA expression levels of EMT markers are affected by OTUB1 in CRC cell lines. OTUB1 was overexpressed or downregulated in SW480 cells (A) or SW620 cells (B), respectively, and the mRNA expression level of TCF8/ZEB1, E-cadherin, β-catenin, and vimentin was detected by q-PCR. β-actin was used as an endogenous control (**P < 0.01, * *P* < 0.05). (PNG 478 KB)

Additional file 10: Figure S8: OTUB1 regulates the PI3K-AKT-GSK3β signaling pathway. After overexpressing OTUB1 in SW480 cells or knocking down OTUB1 in SW620 cells, PTEN, p-AKT, AKT, p-GSK3β (S9), and GSK3β were measured by Western blot (A). After LY294002 or DMSO treatment for 2 hours, p-AKT, AKT, p-GSK3β (S9), GSK3β, E-cadherin, β-catenin and vimentin were measured in SW480-OTUB1 and SW480-Control cells by western blot (B). (PNG 1017 KB)

Additional file 11: Figure S9: OTUB1 does not affect expression of TCF1, LEF1 and DVL2. After overexpressing OTUB1 in SW480 or DLD-1 cells or knocking down OTUB1 in SW620 cells, the protein expression level of TCF1, LEF1 and DVL2 were measured by Western blot. (PNG 199 KB)

Additional file 12: Figure S10: OTUB1 does not affect MAKP signaling path. After overexpressing OTUB1 in SW480 or DLD-1 cells or knocking down OTUB1 in SW620 cells, the protein expression level of p-JNK, p-ERK, p-p38 (MAPK) were measured by Western blot. (PNG 182 KB)

Additional file 13: Figure S11: OTUB1 regulate NF-κB signaling path. After overexpressing OTUB1 in SW480 or DLD-1 cells or knocking down OTUB1 in SW620 cells, cell, cytoplasmic and nuclear lysate was achieved. The protein expression level of NF-κB and p-IκBα were measured by Western blot. (PNG 478 KB)

Additional file 14: Figure S12: Overexpression of OTUB1 promotes CRC liver metastasis in vivo. SW480-OTUB1 or SW480-Control cells were injected into the tail veins of nude mice. Ten weeks later, the mice were sacrificed. (A) Representative figures of general livers are shown, and metastatic nodules are indicated with red arrows. (B) Representative results for HE and IHC staining of metastatic nodules in the livers are shown. The metastatic nodules are indicated with red arrows. The scale bar represents 50 μm. The statistical analysis is shown in (C) (n=8; * *P* < 0.05). (PNG 513 KB)

Additional file 15: Table S3: Primer sequences in qPCR analysis. (DOCX 14 KB)

## Abbreviations

OTUB1: OTU deubiquitinase, ubiquitin aldehyde binding 1
DUBs: Deubiquitinating enzymes
CRC: Colorectal cancer
OS: Overall survival
PFS: Progression-free survival
CEA: Carcinoembryonic antigen
EMT: Epithelial-mesenchymal transition
IHC: Immunohistochemistry
qPCR: Quantitative real-time PCR
NC: Negative control.
