# Supplementary figures and images for: OTUB1 promotes metastasis and serves as a marker of poor prognosis in colorectal cancer
Source: Mol Cancer. 2014 Nov 28;13:258. doi: 10.1186/1476-4598-13-258 (PMC4351937; doi:10.1186/1476-4598-13-258)

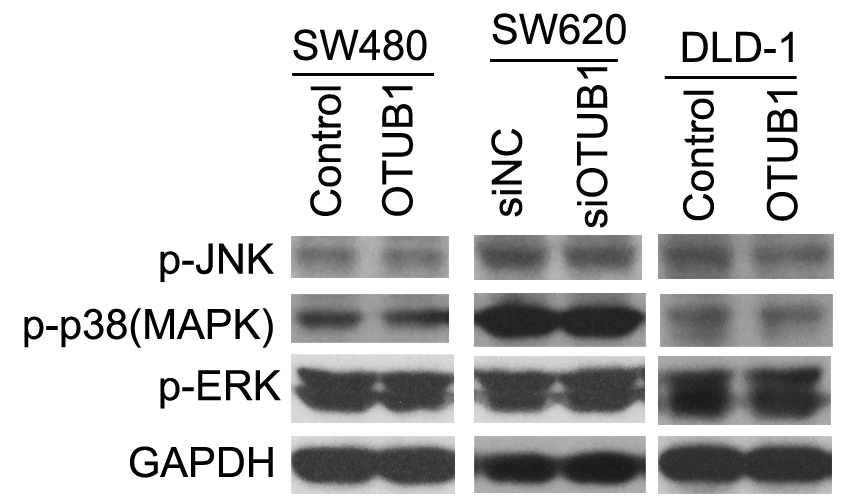

Supplement: Supplementary file 1 — Additional file 1: Figure S1: Representative images (200×magnification) of IHC staining for OTUB1 in CRC tissues. OTUB1 protein expression was scored from 0 to 3. A score of 0 represents negative staining (A), a score of 1 indicates weak positive staining (B), a score of 2 indicates moderate positive staining (C), and a score of 3 represents strong positive staining (D). The scale bar represents 50 μm. (PNG 427 KB) [file 12943_2014_1464_MOESM1_ESM.png]

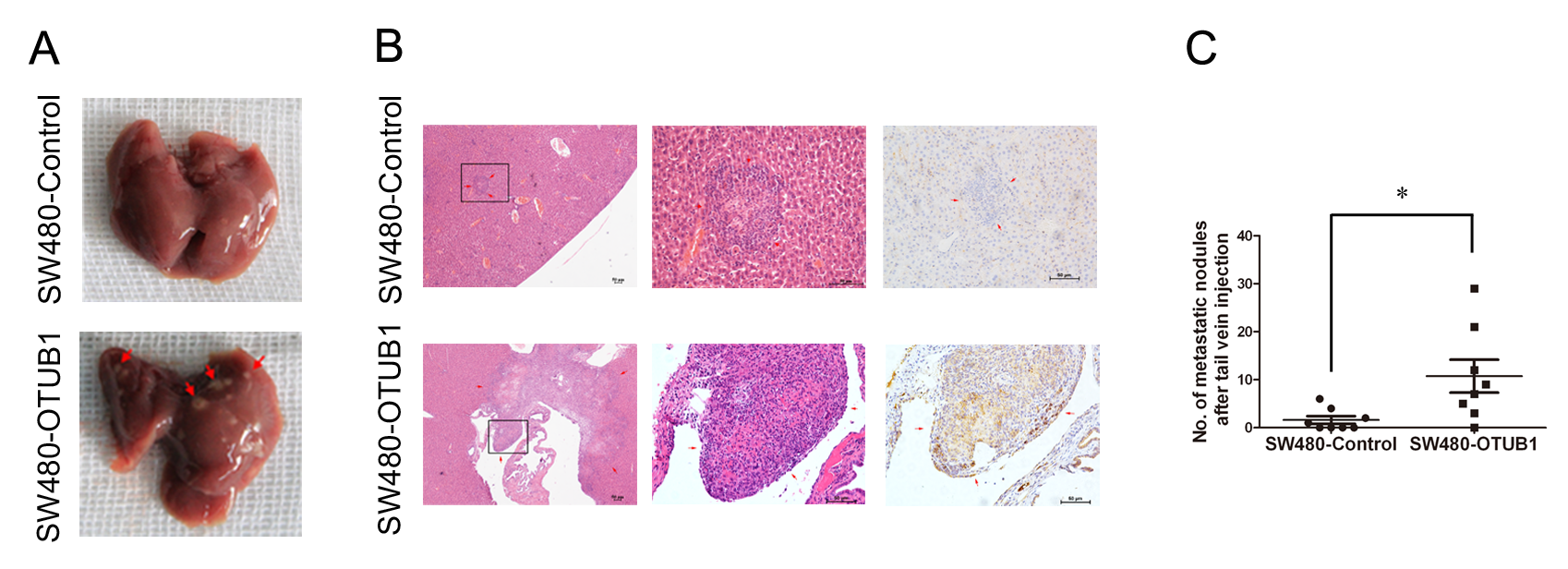

Supplement: Supplementary file 3 — Additional file 3: Figure S2: Kaplan-Meier survival analysis of the correlation between OTUB1 expression and PFS and OS of per stage. (A) Kaplan-Meier method was used to analyze the correlation between OTUB1 expression and PFS and OS of stage I CRC. (B) Kaplan-Meier method was used to analyze the correlation between OTUB1 expression and PFS and OS of stage II CRC. (C) Kaplan-Meier method was used to analyze the correlation between OTUB1 expression and PFS and OS of stage III CRC. (D) Kaplan-Meier method was used to analyze the correlation between OTUB1 expression and PFS and OS of stage IV CRC. (PNG 2 MB) [file 12943_2014_1464_MOESM3_ESM.png]

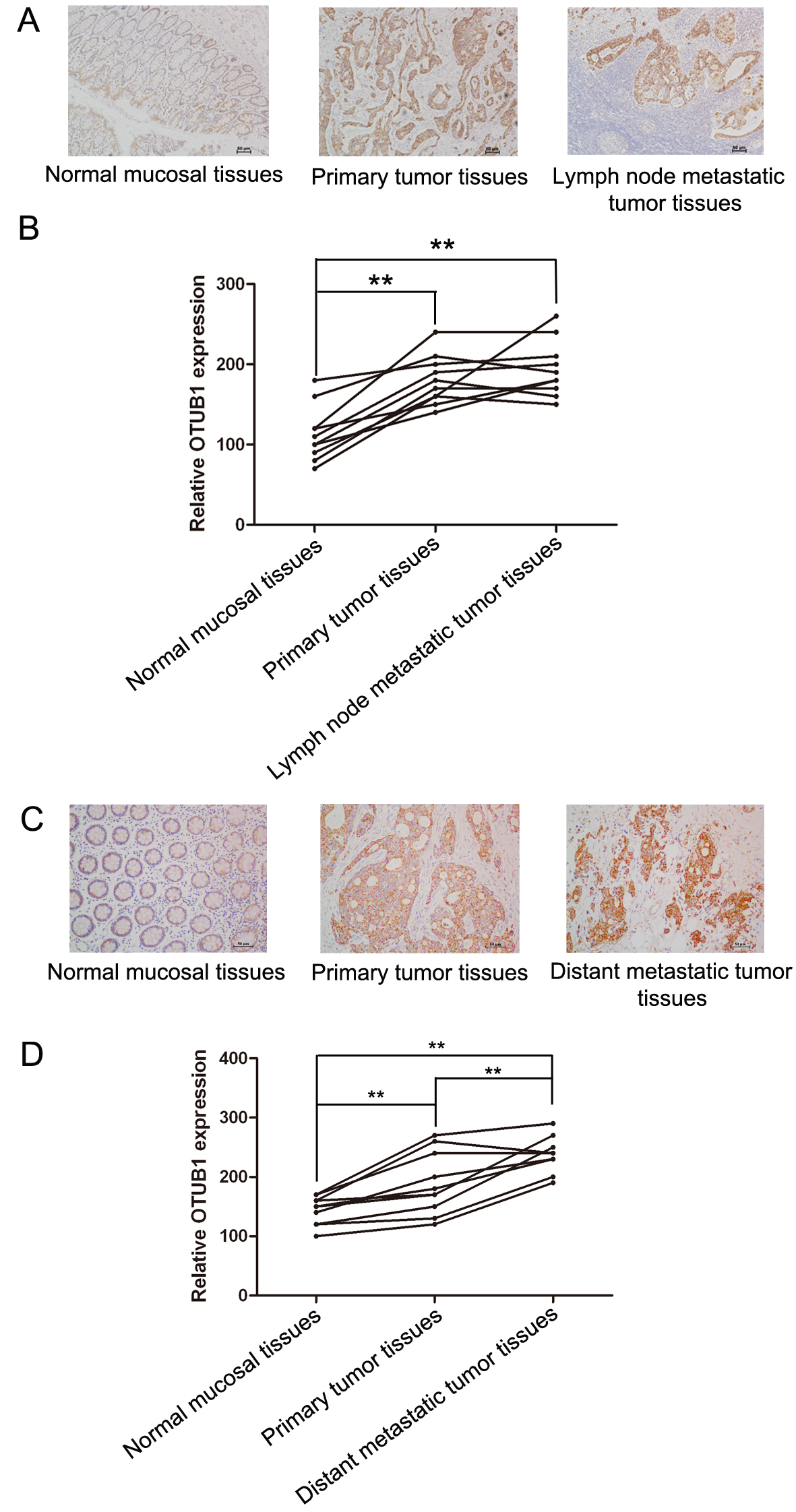

Supplement: Supplementary file 4 — Additional file 4: Figure S3: OTUB1 are expressed in 10 paired adjacent normal mucosal tissues, primary tumor tissues and lymph node metastatic tissues or distant metastatic tissues. (A) Representative images of adjacent normal mucosal tissues, primary tumor tissues and lymph node metastatic tumor tissues from one patient sample are shown. (B) Relative IHC staining for OTUB1 in adjacent normal mucosal tissues, primary tumor tissues, and lymph node metastatic tumor tissues is shown (n=10, **P < 0.01). (C) Representative images of adjacent normal mucosal tissues, primary tumor tissues, and distant metastatic tumor tissues from one patient sample are shown. (D) Relative IHC staining for OTUB1 in adjacent normal mucosal tissues, primary tumor tissues, and distant metastatic tissues is shown (n=10, **P < 0.01, * P < 0.05). (PNG 3 MB) [file 12943_2014_1464_MOESM4_ESM.png]

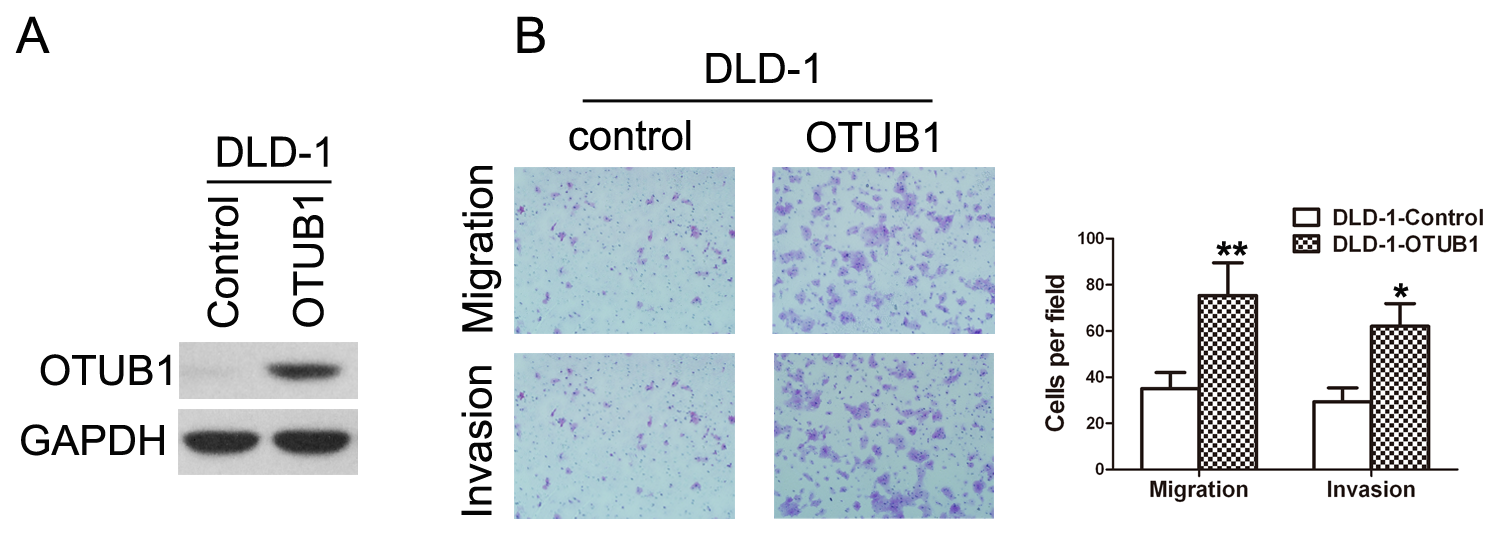

Supplement: Supplementary file 6 — Additional file 6: Figure S4: OTUB1 promotes DLD-1 cells migration and invasion. DLD-1 cells were transfected with the OTUB1 expression plasmid or empty vector for 48 hours, and the expression of OTUB1 at the protein level was examined by Western blot in the OTUB1 overexpression group (DLD-1-OTUB1) and the control group (DLD-1-Control) (A). Representative images showing the migration and invasion of DLD-1-OTUB1 and DLD-1-Control cells are shown (B). The number of tumor cells is quantified on the right. All data are expressed as the means of three independent experiments (**P < 0.01, *P < 0.05). (PNG 1017 KB) [file 12943_2014_1464_MOESM6_ESM.png]

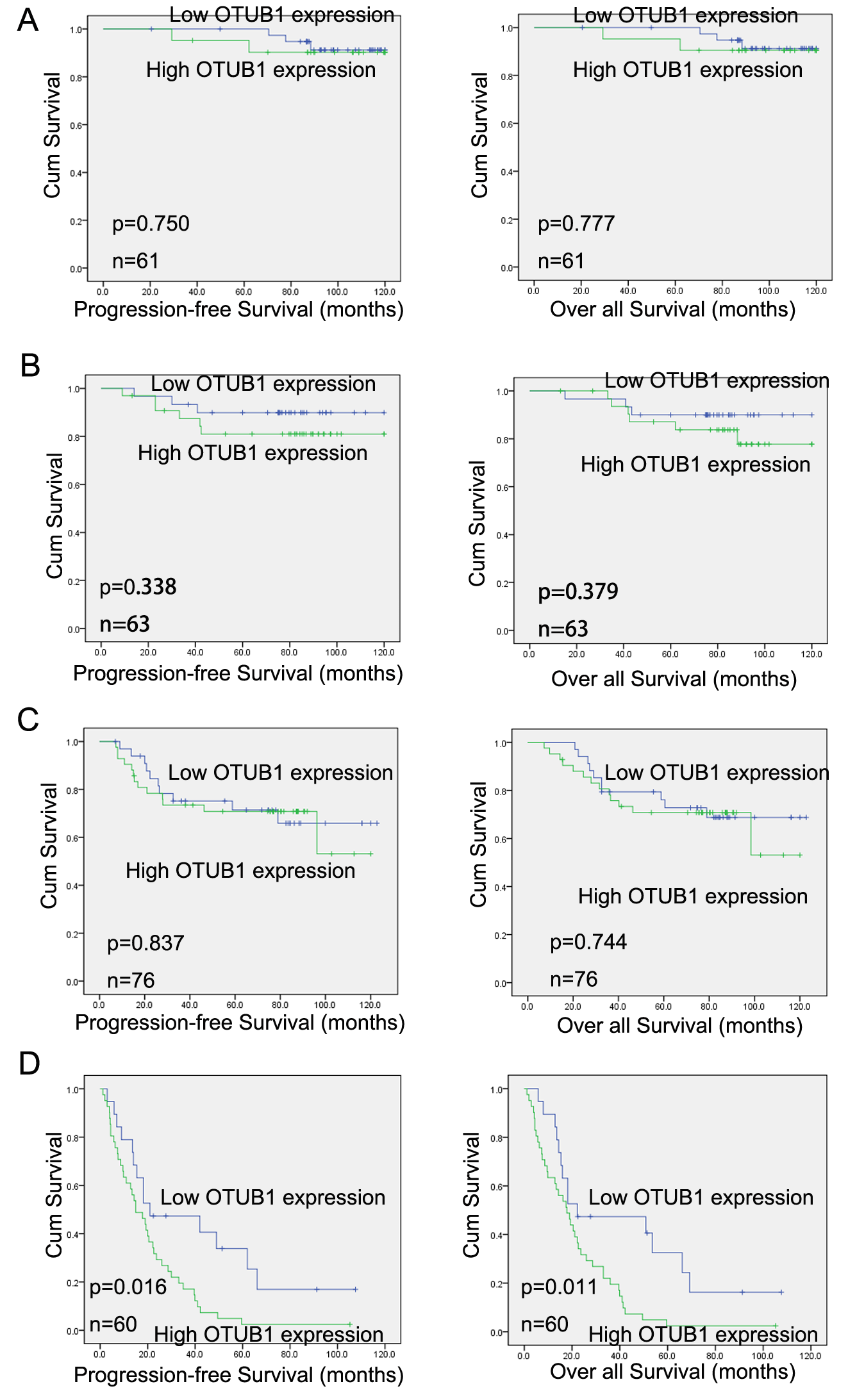

Supplement: Supplementary file 7 — Additional file 7: Figure S5: OTUB1 does not affect the growth of SW480, SW620 and DLD-1. After transfecting SW480, DLD-1 cells with OTUB1 expression plasmid or empty vector or transfecting SW620 cells with siRNA of OTUB1 or NC, the cell growth rate were detected at 0, 24, 48, 72, 96 hours. A-C represented SW480, SW620 and DLD-1 cells respectively. (PNG 949 KB) [file 12943_2014_1464_MOESM7_ESM.png]

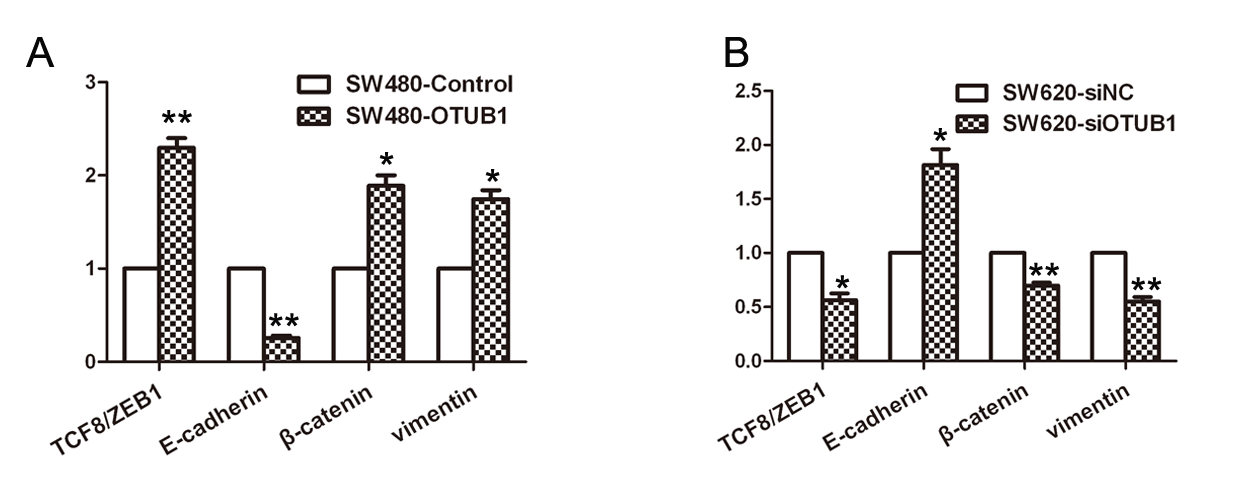

Supplement: Supplementary file 9 — Additional file 9: Figure S7: The mRNA expression levels of EMT markers are affected by OTUB1 in CRC cell lines. OTUB1 was overexpressed or downregulated in SW480 cells (A) or SW620 cells (B), respectively, and the mRNA expression level of TCF8/ZEB1, E-cadherin, β-catenin, and vimentin was detected by q-PCR. β-actin was used as an endogenous control (**P < 0.01, * P < 0.05). (PNG 478 KB) [file 12943_2014_1464_MOESM9_ESM.png]

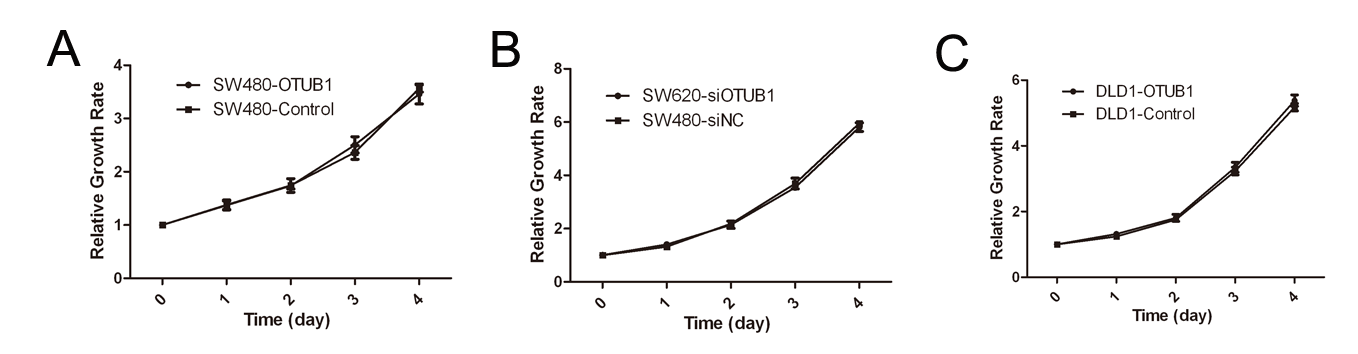

Supplement: Supplementary file 11 — Additional file 11: Figure S9: OTUB1 does not affect expression of TCF1, LEF1 and DVL2. After overexpressing OTUB1 in SW480 or DLD-1 cells or knocking down OTUB1 in SW620 cells, the protein expression level of TCF1, LEF1 and DVL2 were measured by Western blot. (PNG 199 KB) [file 12943_2014_1464_MOESM11_ESM.png]

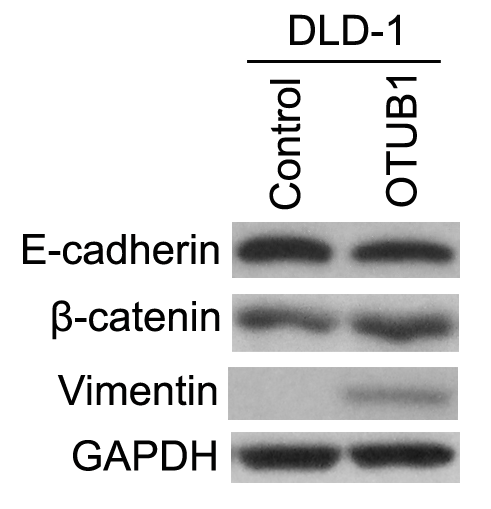

Supplement: Supplementary file 12 — Additional file 12: Figure S10: OTUB1 does not affect MAKP signaling path. After overexpressing OTUB1 in SW480 or DLD-1 cells or knocking down OTUB1 in SW620 cells, the protein expression level of p-JNK, p-ERK, p-p38 (MAPK) were measured by Western blot. (PNG 182 KB) [file 12943_2014_1464_MOESM12_ESM.png]

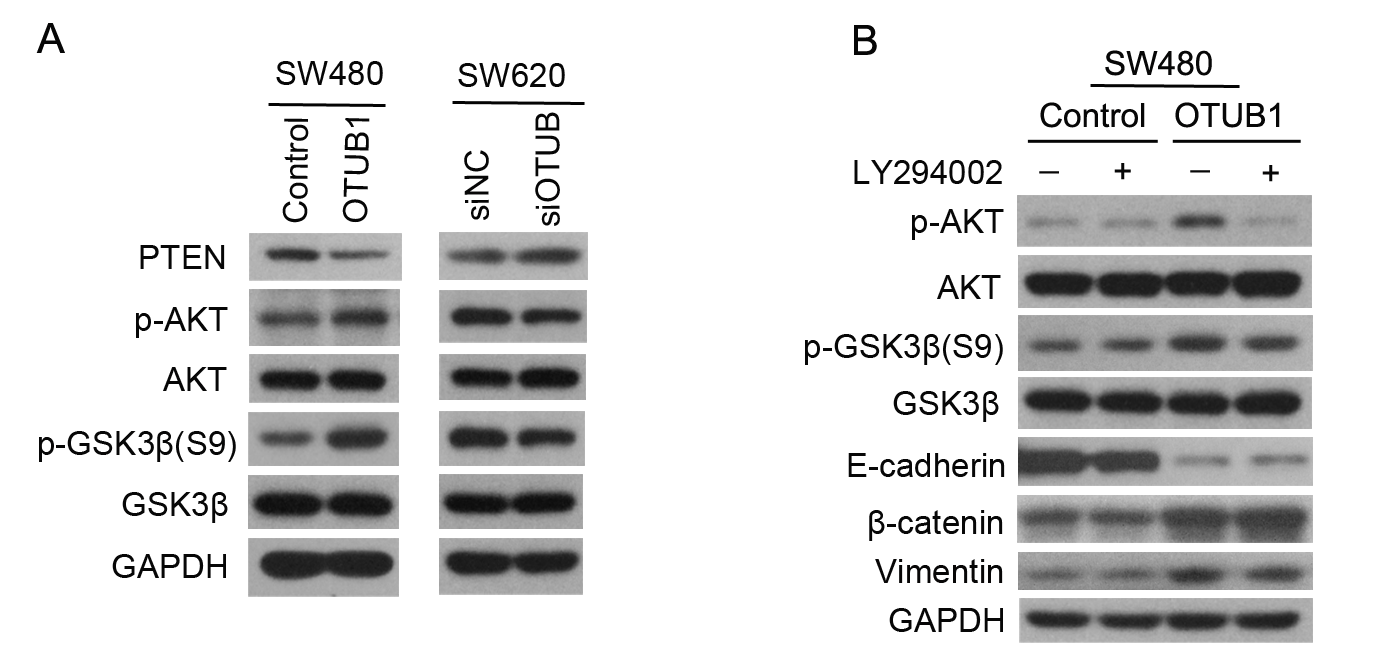

Supplement: Supplementary file 14 — Additional file 14: Figure S12: Overexpression of OTUB1 promotes CRC liver metastasis in vivo. SW480-OTUB1 or SW480-Control cells were injected into the tail veins of nude mice. Ten weeks later, the mice were sacrificed. (A) Representative figures of general livers are shown, and metastatic nodules are indicated with red arrows. (B) Representative results for HE and IHC staining of metastatic nodules in the livers are shown. The metastatic nodules are indicated with red arrows. The scale bar represents 50 μm. The statistical analysis is shown in (C) (n=8; * P < 0.05). (PNG 513 KB) [file 12943_2014_1464_MOESM14_ESM.png]
