# Supplementary material for: OTUB1 promotes metastasis and serves as a marker of poor prognosis in colorectal cancer
Source: Mol Cancer. 2014 Nov 28;13:258. doi: 10.1186/1476-4598-13-258 (PMC4351937; doi:10.1186/1476-4598-13-258)
Supplement: Supplementary file 2 — Additional file 2: Table S1: Chemotherapy of per stage correlation with OTUB1 expression. (DOCX 19 KB) [file 12943_2014_1464_MOESM2_ESM.docx]

| **Additional file 2: Table S1. Chemotherapy of per stage correlation with OTUB1 expression** | | | | | |
| --- | --- | --- | --- | --- | --- |
| **Variables** | **No .(%)** | **OTUB1 low** | | **OTUB1 high** | **P value** |
| **Ⅰ stage 61** | | **40 (65.6)** | | **21(34.4)** | **0.556** |
| **Yes** | **12 (17.9)** | **7 (11.5)** | | **5 (8.2)** |  |
| **No** | **49 (80.3)** | **33 (54.1 )** | | **16 (26.2)** |  |
| **Ⅱ stage** | **63** | **30 (47.6)** | | **33 (50.7)** | **0.269** |
| **Yes** | **34 (54.0)** | **14 (22.2)** | | **20 (31.7)** |  |
| **No** | **29 (46.0)** | **16 (25.4)** | | **13 (20.6)** |  |
| **Ⅲ stage** | **76** | **34 (44.7)** | | **42 (55.3)** | **0.790** |
| **Yes** | **57 (75.0)** | **26 (34.2)** | | **31 (40.8)** |  |
| **No** | **19 (25.0)** | **8 (10.5)** | | **11 (14.5)** |  |
| **Ⅳ stage** | **60** | **19 (31.7)** | | **41 (68.3)** | **0.985** |
| **Yes** | **38 (63.3)** | **12 (20.0)** | | **26 (43.3)** |  |
| **No** | **22 (36.7)** | **7 (11.7)** | | **15 (25.0)** |  |
| *** Statistically significant, *P* < 0.05** | | |  |  |  |
