# Supplementary material for: OTUB1 promotes metastasis and serves as a marker of poor prognosis in colorectal cancer
Source: Mol Cancer. 2014 Nov 28;13:258. doi: 10.1186/1476-4598-13-258 (PMC4351937; doi:10.1186/1476-4598-13-258)
Supplement: Supplementary file 5 — Additional file 5: Table S2a: The H scores of OTUB1 expression in normal mucosal tissues, primary tumor tissues and lymph node metastatic tumor tissues in 10 paired tissues. And Table S2b The H scores of OTUB1 expression in normal mucosal tissues, primary tumor tissues and distant metastatic tumor tissues in 10 paired tissues. (DOCX 17 KB) [file 12943_2014_1464_MOESM5_ESM.docx]

| **Additional file 5: Table S2a. the H scores of OTUB1 expression in normal mucosal tissues,** | | | |
| --- | --- | --- | --- |
| **primary tumor tissues and lymph node metastatic tumor tissues in 10 paired tissues** | | | |
| **Patients** | **Normal mucosal tissues** | **Primary tumor tissues** | **Lymph node metastatic tumor tissues** |
| **NO.1** | **100** | **180** | **160** |
| **NO.2** | **80** | **170** | **170** |
| **NO.3** | **120** | **240** | **240** |
| **NO.4** | **90** | **160** | **260** |
| **NO.5** | **120** | **150** | **180** |
| **NO.6** | **70** | **160** | **150** |
| **NO.7** | **180** | **200** | **210** |
| **NO.8** | **110** | **190** | **200** |
| **NO.9** | **100** | **140** | **180** |
| **NO.10** | **160** | **210** | **190** |
|  |  |  |  |
|  |  |  |  |
| **S2b. the H scores of OTUB1 expression in normal mucosal tissues, primary tumor tissues** | | | |
| **and distant metastatic tumor tissues in 10 paired tissues** | | | |
| **Patients** | **Normal mucosal tissues** | **Primary tumor tissues** | **Distant metastatic tumor tissues** |
| **NO.1** | **120** | **130** | **200** |
| **NO.2** | **160** | **260** | **240** |
| **NO.3** | **140** | **200** | **230** |
| **NO.4** | **100** | **120** | **190** |
| **NO.5** | **150** | **170** | **270** |
| **NO.6** | **160** | **170** | **270** |
| **NO.7** | **170** | **270** | **290** |
| **NO.8** | **150** | **180** | **230** |
| **NO.9** | **120** | **150** | **250** |
| **NO.10** | **170** | **240** | **240** |
