# Supplementary material for: OTUB1 promotes metastasis and serves as a marker of poor prognosis in colorectal cancer
Source: Mol Cancer. 2014 Nov 28;13:258. doi: 10.1186/1476-4598-13-258 (PMC4351937; doi:10.1186/1476-4598-13-258)
Supplement: Supplementary file 15 — Additional file 15: Table S3: Primer sequences in qPCR analysis. (DOCX 14 KB) [file 12943_2014_1464_MOESM15_ESM.docx]

| **Additional file 15: Table S3. Primer sequences in qPCR analysis** | | |
| --- | --- | --- |
| **genes** | **primers** | |
| β-actin | Sense | TGGCACCCAGCACAATGAA |
|  | Antisense | CTAAGTCATAGTCCGCCTAGAAGCA |
|  |  |  |
| TCF8/ZEB1 | Sense | AGCTGCCAATAAGCAAACGA |
|  | Antisense | GGCGGTGTAGAATCAGAGTCAT |
|  |  |  |
| E-cadherin | Sense | TGCTCACATTTCCCAACTC |
|  | Antisense | TCTGTCACCTTCAGCCATC |
|  |  |  |
| β-catenin | Sense | CAAGTGGGTGGTATAGAGGCT |
|  | Antisense | GTGGGATGGTGGGTGTAAGAG |
|  |  |  |
| Vimentin | Sense | CTTGAACGCAAAGTGGAATC |
|  | Antisense | GAGGTCAGGCTTGGAAACA |
|  |  |  |
| OTUB1 | Sense | TTTCTATCGGGCTTTCGGA |
|  | Antisense | TCGGAGGTGCTCTGGTCAT |
